# Supplementary figures and images for: SnoRNA Expression and RNA 2’-O-Methylation in Drosophila melanogaster S2 Cells
Source: bioRxiv. 2026 May 22:2026.05.21.726978. Preprint. [Version 1] doi: 10.64898/2026.05.21.726978 (PMC13228274; doi:10.64898/2026.05.21.726978)

Fig. S1

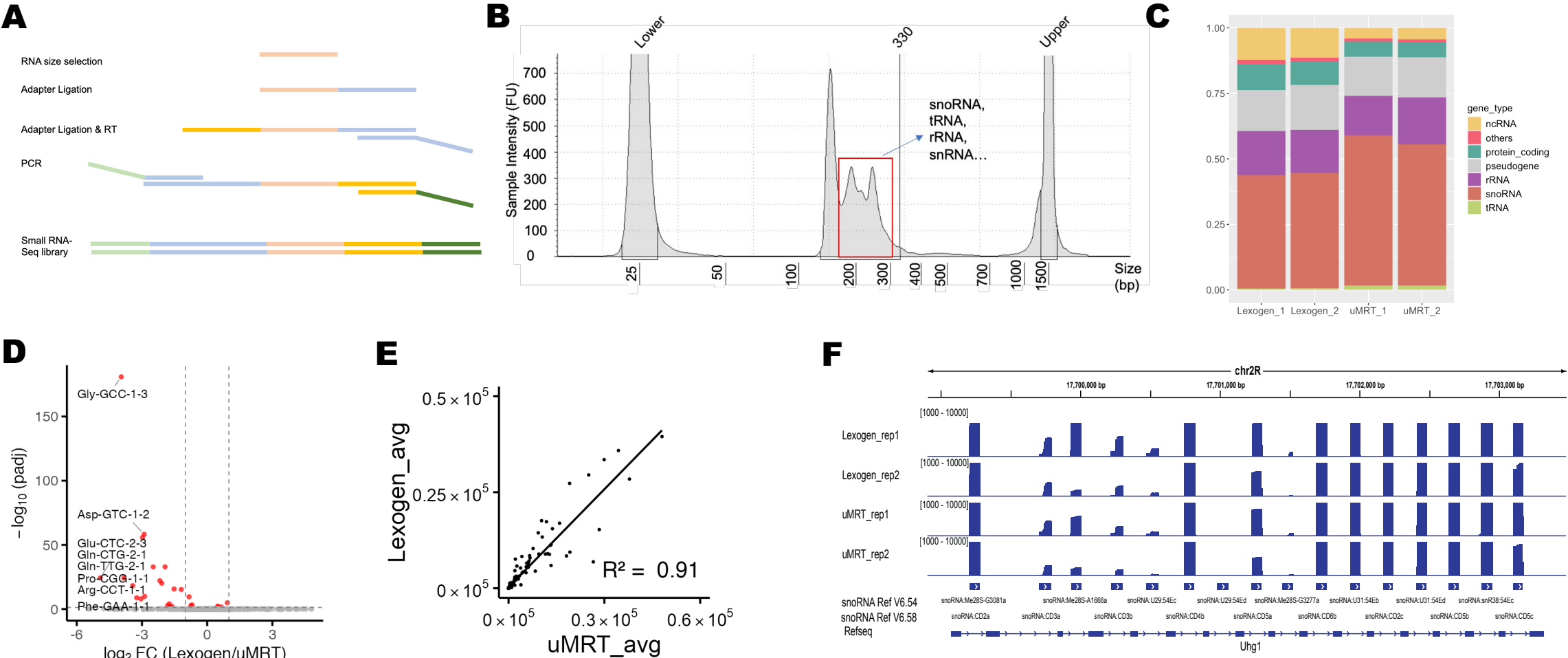

Supplement: Supplement 1 — Figure S1. Small RNA Sequencing Results in Drosophila S2 Cells A. Schematic of small RNA-Seq. The workflow was adapted from the Lexogen Small RNA Library Prep kit. B. Representative tapestation electropherogram of a small RNA-Seq library. Size ranges corresponding to RNA species of interest are highlighted by red boxes. C. Stacked bar plot showing the proportion of detected genes in each biotype across small RNA-seq libraries, color-coded by biotype. D. Volcano plot comparing small RNA-seq libraries prepared with Lexogen RT versus uMRT enzymes. Significantly differentially expressed genes (∣log2FC∣ > 1, p < 0.05) are shown in red, with selected tRNA species labeled. E. Pairwise TPM correlation between small RNA-seq libraries; Pearson correlation coefficients are indicated. F. Representative snoRNA expression profiles from small RNA-Seq data. Multiple genome annotation versions are shown, snoRNA nomenclature differs between annotation releases. [file media-1.pdf]

Fig. S3

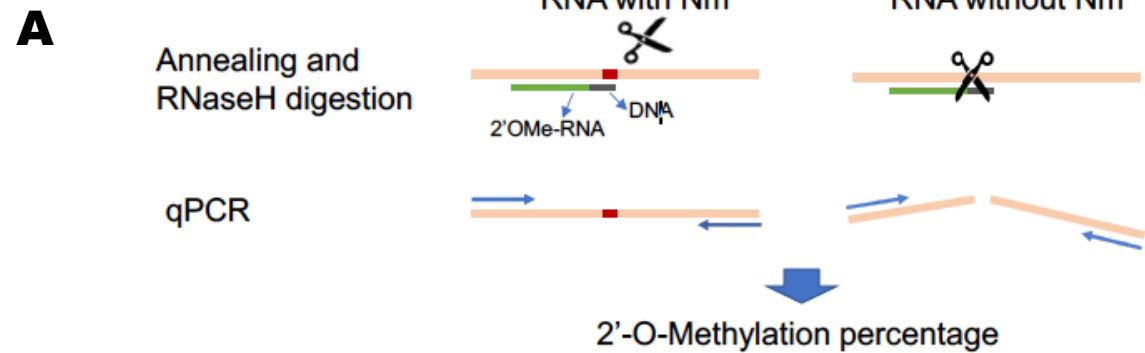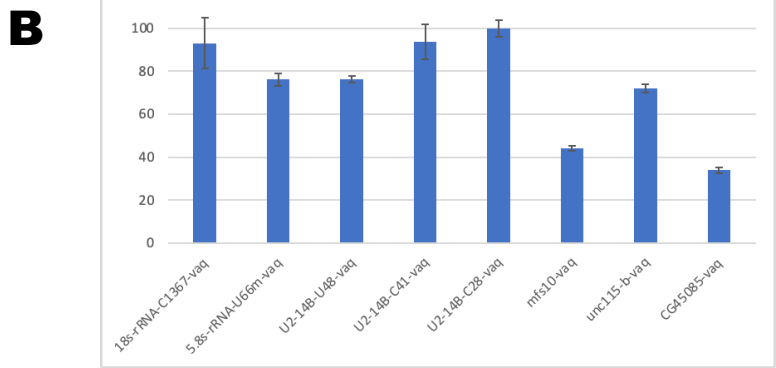

Supplement: Supplement 3 — Figure S3. Quantitation of 2’-O-methylation sites in Drosophila RNAs by Nm-VAQ. A. Schematic of the Nm-VAQ method used for detecting and quantifying 2’-O-methyl (Nm) modifications. The DNA/2’-O-methylated RNA chimeric oligonucleotide targeting the Nm site was annealed to the RNA and treated with RNase H. RNase H cleaves RNA-DNA hybrids at unmethylated sites, and qPCR is used to determine the Nm ratio. B. The methylation ratio in Drosophila RNA Nm sites detected by Nm-VAQ. Error bars represent the standard error for three technical replicates. [file media-3.pdf]

Fig. S4

A

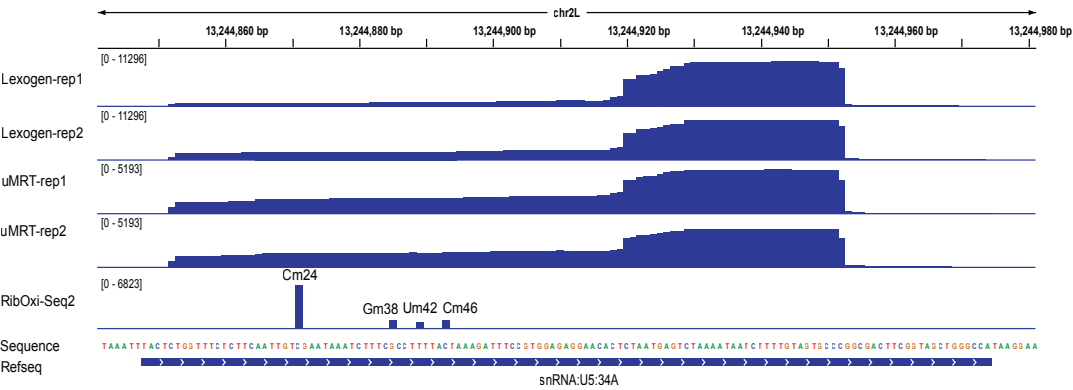

Supplement: Supplement 4 — Figure S4. 2’-O-methylation sites detected in Drosophila U5 snRNA by RibOxi-Seq2. A. Representative view of U5 snRNA showing small RNA-seq expression and RibOxi-seq2 signal. Each RibOxi-seq2 peak corresponds to a previously validated or annotated Nm modification site. [file media-4.pdf]

Fig. S5

A

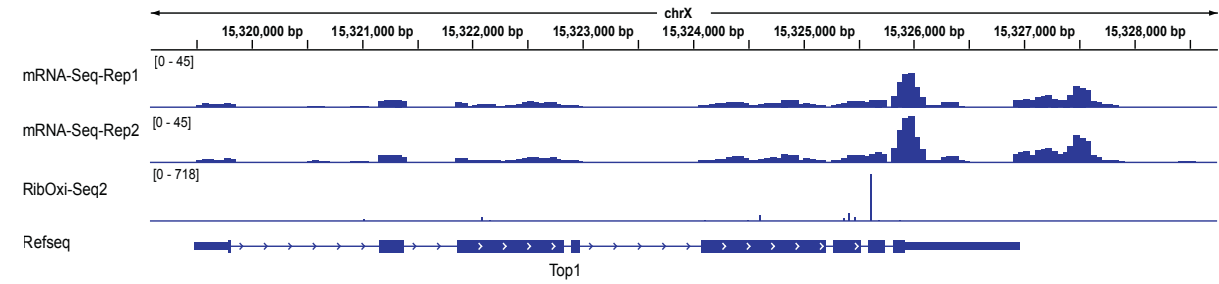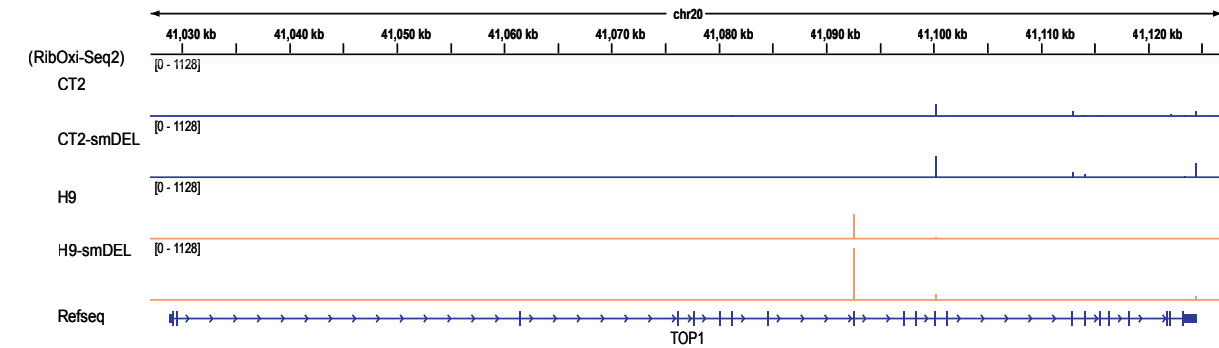

B

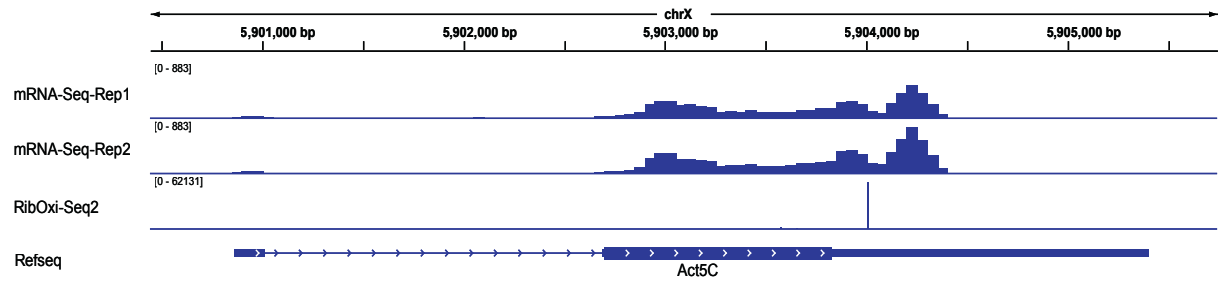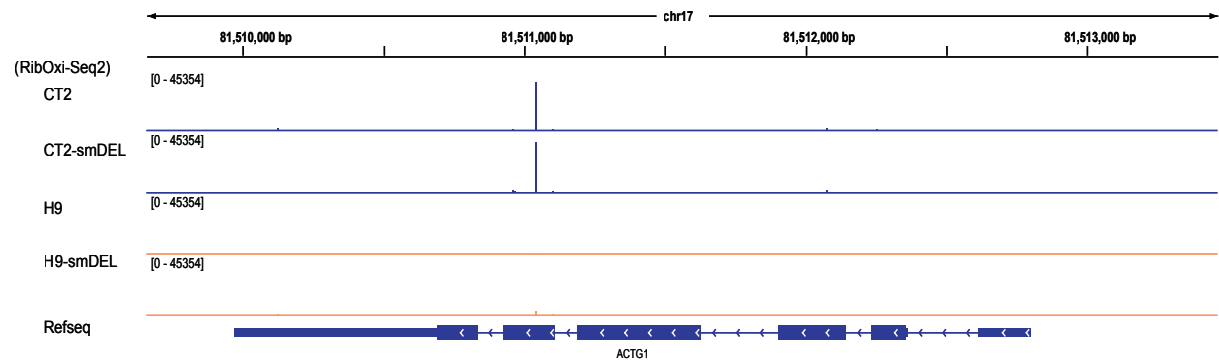

Supplement: Supplement 5 — Figure S5. Detection of 2’-O-methylation sites in Drosophila mRNAs by RibOxi-Seq2. A. Representative RibOxi-seq2 signal tracks at the TOP1 locus in human and Drosophila. B. Representative RibOxi-seq2 signal tracks at the ACTG1 locus in human and Drosophila. [file media-5.pdf]
