# Supplementary material for: SnoRNA Expression and RNA 2’-O-Methylation in Drosophila melanogaster S2 Cells
Source: bioRxiv. 2026 May 22:2026.05.21.726978. Preprint. [Version 1] doi: 10.64898/2026.05.21.726978 (PMC13228274; doi:10.64898/2026.05.21.726978)
Supplement: Supplement 2 — Figure S2. RibOxi-Seq2 detection of 2’-O-methylation in rRNAs in Drosophila S2 cells. A. Secondary structures of Drosophila 18S and 28S rRNAs. Nm sites identified in both RibOxi-seq2 and RiboMeth-seq are highlighted by brown circles; sites detected only by RiboMeth-seq are shown in cyan. B. RibOxi-Seq2 peaks in large subunit of mitochondria rRNA and the 28S rRNA region. Peak aligns with validated or annotated Nm reference. C. Multiple sequence alignment of 18S rRNA between human and Drosophila. Brown highlighting indicates Nm sites identified by RibOxi-seq2 in this study and those previously annotated. D. Multiple sequence alignment of 28S rRNA between human and Drosophila. Brown highlighting indicates Nm sites identified by RibOxi-seq2 in this study and those previously annotated. [file media-2.pdf]

Fig. S2

A

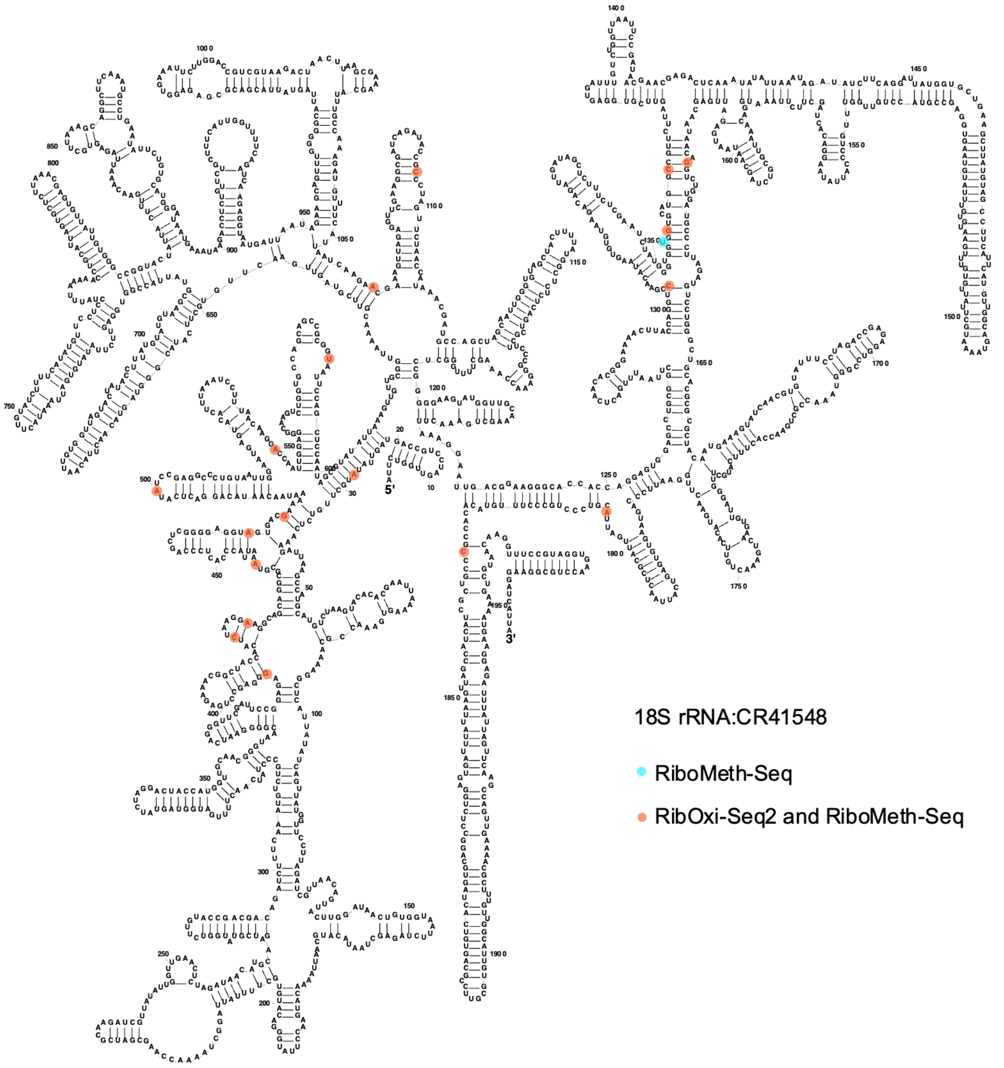
18S rRNA:CR41548
RiboMeth-Seq
RibOxi-Seq2 and RiboMeth-Seq

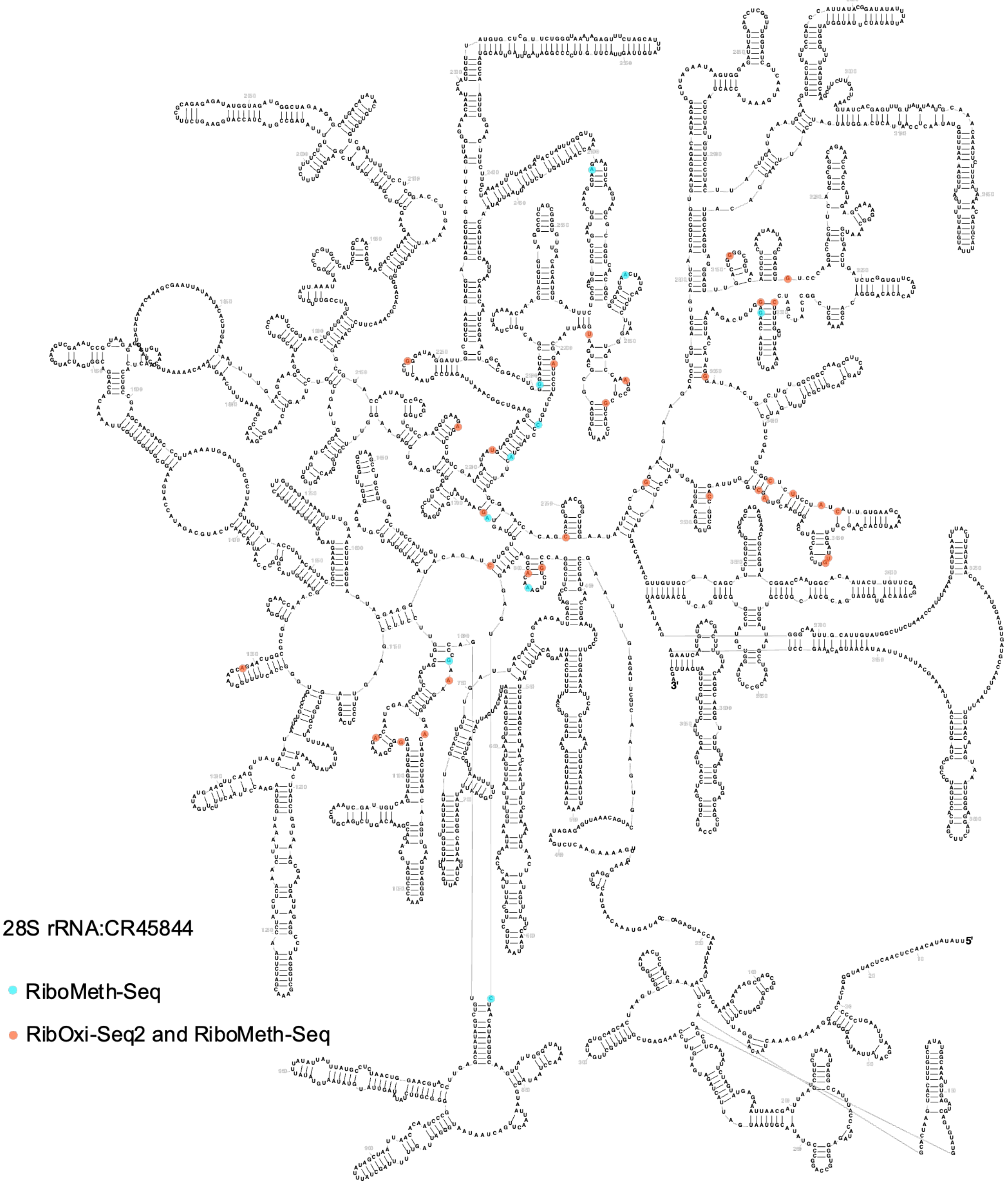
28S rRNA:CR45844
RiboMeth-Seq
RibOxi-Seq2 and RiboMeth-Seq

Fig. S2

B

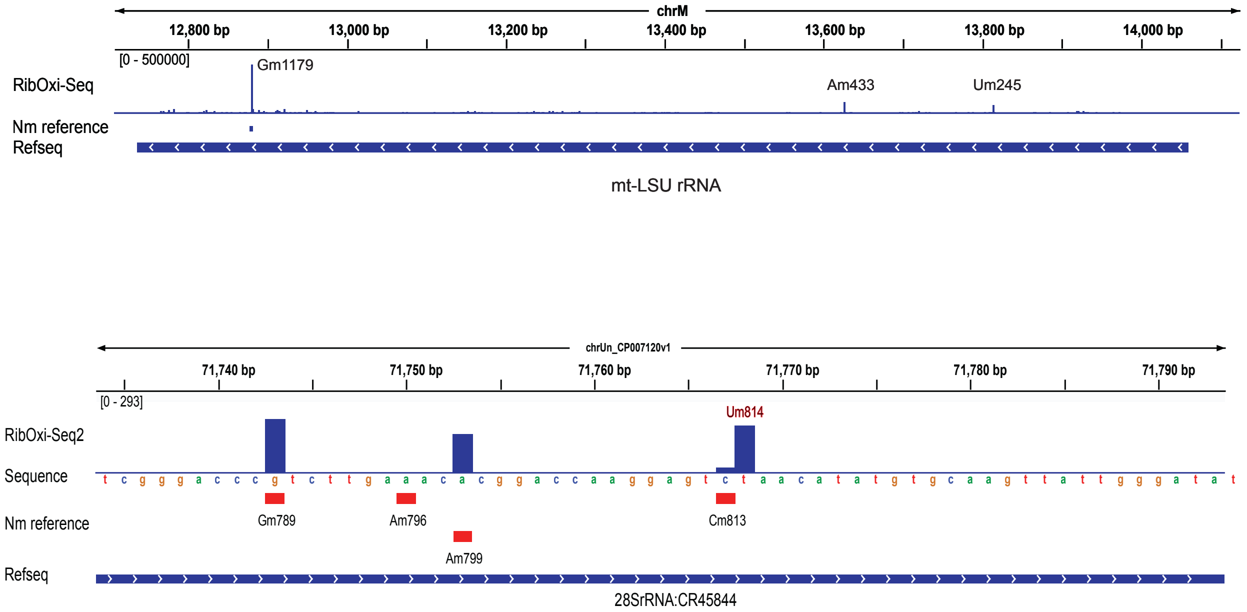
chrM
12,800 bp
13,000 bp
13,200 bp
13,400 bp
13,600 bp
13,800 bp
14,000 bp
[0 - 500000]
RibOxi-Seq
Gm1179
Am433
Um245
Nm reference
Refseq
mt-LSU rRNA
chrUn_CP007120v1
71,740 bp
71,750 bp
71,760 bp
71,770 bp
71,780 bp
71,790 bp
[0 - 293]
RibOxi-Seq2
Um814
Sequence
t c g g g a c c c g t c t t g a a a c a c g g a c c a a g g a g t c t a a c a t a t g t g c a a g t t a t t g g g a t a t
Gm789
Am796
Cm813
Am799
Nm reference
Refseq
28SrRNA:CR45844

Fig S xx

```
RNA18SN5/1-1869          1 UACCUGGUUGAUCCUGCCAGUAG-CAUAUGCUUGUCUCAAAGAUUAAGCCAUGCAUGUCUGAGUACG 66
18SRNA:CR41548/2-1995    2 UU-CUGGUUGAUCCUGCCAGUAGUUAUAUGCUUGUCUCAAAGAUUAAGCCAUGCAUGUCUAAGUACA 67

RNA18SN5/1-1869         67 CACGGCCGGU-ACAGUGAAACUGCGAAUGGCUCAUUAAAUCAGUUAUGGUUCCUUUGGUCGCU--C- 129
18SRNA:CR41548/2-1995   68 CACGAAU--UAAAAGUGAAACCGCAAAAGGCUCAUUAUAUCAGUUAUGGUUCCUUAGAUCGUUAACA 132

RNA18SN5/1-1869        130 GCUCCUCUCCUACUUGGAUAACUGUGGUAAUUCUAGAGCUAAUACAUGC-----CGACGGGCGCUGA 191
18SRNA:CR41548/2-1995  133 G------U--UACUUGGAUAACUGUGGUAAUUCUAGAGCUAAUACAUGCAAUUAAAACA-----UGA 186

RNA18SN5/1-1869        192 CCCCCUUCGCGGGGGGGAUGCGUGCAUUUAUCA-GAUCAAAACCAACCCGGUCAGCCCCUCUCCGGC 257
18SRNA:CR41548/2-1995  187 ACC--UUAU---GGGACAUG--UGCUUUUAUUAGGCU-AAAACCAA--------------------- 224

RNA18SN5/1-1869        258 CCCGGCCGGGGGGCGGGCGCCGG--CG-GCU-UUGG-UG-ACUCUAGAUAACCUCGGGCCGAUCGCA 318
18SRNA:CR41548/2-1995  225 ------------GCGAUCGCAAGAUCGUUAUAUUGGUUGAACUCUAGAUAACAU---GCAGAUCGUA 276

RNA18SN5/1-1869        319 CGCCCCCCGUGG-C--G---GCGACGAC---CCAUUCGAACGUCUGCCCUAUCAACUUUCGAUGGUA 376
18SRNA:CR41548/2-1995  277 ---------UGGUCUUGUAC-CGACGACAGAUCUUUCAAAUGUCUGCCCUAUCAACUUUUGAUGGUA 333

RNA18SN5/1-1869        377 GUCGCCGU--GCCUACCAUGG-UGACCACGGGUGACGGGGAAUCAGGGUUCGAUUCCGGAGAGGGAG 440
18SRNA:CR41548/2-1995  334 GUAUC--UAGGACUACCAUGGUUG-CAACGGGUAACGGGGAAUCAGGGUUCGAUUCCGGAGAGGGAG 397

RNA18SN5/1-1869        441 CCUGAGAAACGGCUACCACAUCCAAGGAAGGCAGCAGGCGCGCAAAUUACCCACUCCC-GACCCGGG 506
18SRNA:CR41548/2-1995  398 CCUGAGAAACGGCUACCACAUCUAAGGAAGGCAGCAGGCGCGUAAAUUACCCACUCCCAG-CUCGGG 463

RNA18SN5/1-1869        507 GAGGUAGUGACGAAAAAUAACAAUACAGGACUC-U-UUCGAGGCCCUGUAAUUGGAAUGAGUCCACU 571
18SRNA:CR41548/2-1995  464 GAGGUAGUGACGAAAAAUAACAAUACAGGACUCAUAUCCGAGGCCCUGUAAUUGGAAUGAGUACACU 530

RNA18SN5/1-1869        572 UUAAAUCCUUUAACGAGGAUCC-AUUGGAGGGCAAGUCUGGUGCCAGCAGCCGCGGUAAUUCCAGCU 637
18SRNA:CR41548/2-1995  531 UUAAAUCCUUUAACAAGGA-CCAAUUGGAGGGCAAGUCUGGUGCCAGCAGCCGCGGUAAUUCCAGCU 596

RNA18SN5/1-1869        638 CCAAUAGCGUAUAUUAAAGUUGCUGCAGUUAAAAAGCUCGUAGUUGGAUCUUGGG------AGCGGG 698
18SRNA:CR41548/2-1995  597 CCAAUAGCGUAUAUUAAAGUUGUUGCGGUUAAAACGUUCGUAGUUGAA-CUUGUGCUUCAUA-CGGG 661

RNA18SN5/1-1869        699 ------------C----G-GGCG-GUCCG---CC-----G--CG-AGGCG-AGC-CACCGCCCGUCC 734
18SRNA:CR41548/2-1995  662 UAGUACAACUUACAAUUGUGGUUAGUACUAUACCUUUAUGUAUGUAAGCGUA--UUACCG---GUG- 722

RNA18SN5/1-1869        735 CCG----C---------------CCCUUG---CCUCUC----G---GCGCCC---------CCU-CG 762
18SRNA:CR41548/2-1995  723 --GAGUUCUUAUAUGUGAUUAAAUACUUGUAUCUUUUCAUAUGUUC-CUCCUAUUUAAAAACCUGCA 786

RNA18SN5/1-1869        763 --A-UGCUCUU-AGCUGAGUGUCCCGCG-GGGCCCGAAGCGU-UUACUUUGAAAAAAUUAGAGUG-U 822
18SRNA:CR41548/2-1995  787 UUAGUGCUCUUAAAC-GAGUGUUAUU-GUGGGCCGGUA-C-UAUUACUUUGAACAAAUUAGAGUGCU 849

RNA18SN5/1-1869        823 UCAAAGCAGGC-CCGAGCCGCCUGGAUA--C--CGCAGCUAGGAAUAAUGGAAUAGGACCGCGGUUC 884
18SRNA:CR41548/2-1995  850 U-AAAGCAGGCUUCAAAU-GCCUGAAUAUUCUGUGCA--UGGGA-UAAUGAAAUAAGACCUCUGUUC 911

RNA18SN5/1-1869        885 U-AUUUUGUUGGUUUUCGGAAC-UGAGGCCAUGAUUA--AGAGGGACGGCC-GGGGGCAUUCGUAUU 946
18SRNA:CR41548/2-1995  912 UGCUUUCAUUGGUUUUCAGAUCAAGAGGUAAUGAUUAAUAGAAGCA--GUUUGGGGGCAUUAGUAUU 976

RNA18SN5/1-1869        947 GCGCCGCUAGAGGUGAAAUUCUUGGACCGGCGCAAGACGGAC-CAGAGCGAAAGCAUUUGCC-AAGA 1011
18SRNA:CR41548/2-1995  977 ACGACGCGAGAGGUGAAAUUCUUGGACCGUCGUAAGACUAACUUA-AGCGAAAGCAUUUGCCAAAGA 1042

RNA18SN5/1-1869       1012 AUGUUUUCAUUAAUCAAGAACGAAAGUCGGAGGUUCGAAGACGAUCAGAUACCGUCGUAGUUCCGAC 1078
18SRNA:CR41548/2-1995 1043 -UGUUUUCAUUAAUCAAGAACGAAAGUUAGAGGUUCGAAGGCGAUCAGAUACCGCCCUAGUUCUAAC 1108

RNA18SN5/1-1869       1079 CAUAAACGAUGCC-GACCGGCGA-UGCGGCG--GCGUUA-UUCCCAUGACCCGC-CGGGCAGCUU-C 1138
18SRNA:CR41548/2-1995 1109 CAUAAACGAUGCCAG-CUAGCAAUUG-GGUGUAGC--UACUUUU-AUGGCUCUCUCAGUC-GCUUCC 1169

RNA18SN5/1-1869       1139 CGGGAAACCAAAG-UCUUUGGGUUCCGGGGGGAGUAUGGUUGCAAAGCUGAAACUUAAAGGAAUUGA 1204
18SRNA:CR41548/2-1995 1170 CGGGAAACCAAAGCU-UUUGGGCUCCGGGGGAAGUAUGGUUGCAAAGCUGAAACUUAAAGGAAUUGA 1235

RNA18SN5/1-1869       1205 CGGAAGGGCACCACCAGGAGUGGAGCCUGCGGCUUAAUUUGACUCAACACGGGAAACCUCACCCGGC 1271
18SRNA:CR41548/2-1995 1236 CGGAAGGGCACCACCAGGAGUGGAGCCUGCGGCUUAAUUUGACUCAACACGGGAAACUUACCAGGU 1302

RNA18SN5/1-1869       1272 CCGGACACGGACAG---GAUU-GACAGAUUGAUAGCUCUUUCUCGAUUCCGUGGGUGGUGGUGCAUG 1334
18SRNA:CR41548/2-1995 1303 CCGAACAU--A-AGUGUG-UAAGACAGAUUGAUAGCUCUUUCUCGAAUCUAUGGGUGGUGGUGCAUG 1365

RNA18SN5/1-1869       1335 GCCGUUCUUAGUUGGUGGAGCGAUUUGUCUGGUUAAUUCCGAUAACGAACGAGACUC---------- 1391
18SRNA:CR41548/2-1995 1366 GCCGUUCUUAGUUCGUGGAGUGAUUUGUCUGGUUAAUUCCGAUAACGAACGAGACUCAAAUAUAUUA 1432

RNA18SN5/1-1869       1392 -------------------------------------------------------UGGC------AUGC 1399
18SRNA:CR41548/2-1995 1433 AAUAGAUAUCUUCAGGAUUAUGGUGCUGAAGCUUAUGUAGCCUUCAUUCAUGUUGGCAGUAAAAUGC 1499

RNA18SN5/1-1869       1400 UAACU--------A---GU-----UACG-CGACCCCCG-AGCGGUCGG--CGUCCC----------- 1435
18SRNA:CR41548/2-1995 1500 UUAUUGUGUUUGAAUGUGUUUAUGUAAGUGGAGCC--GUACCUGUUGGUUUGUCCCAUUAUAAGGAC 1564

RNA18SN5/1-1869       1436 -CCAACUUCUUAGAGGGACAAGUGGCGU-UCAGC--CACCCGAGAUUGAGCAAUAACAGGUCUGUGA 1498
18SRNA:CR41548/2-1995 1565 ACUAGCUUCUUAAAUGGACAAAUUGCGUCU-AGCAAUAAU-GAGAUUGAGCAAUAACAGGUCUGUGA 1629

RNA18SN5/1-1869       1499 UGCCCUUAGAUGUCCGGGGCUGCACGCGCGCUACACUGACUGGCUCAGCGUGUGCCUA-CCCUACG- 1563
18SRNA:CR41548/2-1995 1630 UGCCCUUAGAUGUCCUGGGCUGCACGCGCGCUACAAUGAAAGUAUCAACGUGUA--UUU-CCUA-GA 1692

RNA18SN5/1-1869       1564 CCG-GCAGG-CGCGGGUAACCCGUUGAACCCCAUUCGUGAUGGGGAUCGGGGAUUGCAAUUAUUCCC 1628
18SRNA:CR41548/2-1995 1693 CCGAG-AGGUC-CGGGUAAACCGCUGAACCACUUUCAUGCUUGGGAUUGUGAACUGAAACUGUUCAC 1757

RNA18SN5/1-1869       1629 CAUGAACGAGGAAUUCCCAGUAAGUGCGGGUCA-UAAGCUUGCGUUGAUUAAGUCCCUGCCCUUUGU 1694
18SRNA:CR41548/2-1995 1758 -AUGAACUUGGAAUUCCCAGUAAGUGUGAGUCAUUAA-CUCGCAUUGAUUACGUCCCUGCCCUUUGU 1822

RNA18SN5/1-1869       1695 ACACACCGCCCGUCGCUACUACCGAUUGGAUGGUUUAGUGAGGCCCUCGGAUCGGCCCCGCCGGGGU 1761
18SRNA:CR41548/2-1995 1823 ACACACCGCCCGUCGCUACUACCGAUUGAAUUAUUUAGUGAGGUC-UC----CGGA-----CGUGAU 1879

RNA18SN5/1-1869       1762 C---G-GCCCACGGCCCU--G-G---CGGA-G---CGCUGAGAA---GACGGUCGAACUUGACUAUC 1811
18SRNA:CR41548/2-1995 1880 CACUGUG---ACG-CCUUGCGUGUUACGGUUGUUUCGC--AAAAGUUGAC---CGAACUUGAUUAUU 1937
```

RNA18SN5/1-1869	1812	UAGAGGAAGUAAAAGUCGUAACAAGGUUUCCGUAGGUGAACCUGCGGAAGGAUCAUUA	1869
18SRNA:CR41548/2-1995	1938	UAGAGGAAGUAAAAGUCGUAACAAGGUUUCCGUAGGUGAACCUGCGGAAGGAUCAUUA	1995

Fig S xxx

```
RNA28SN5/1-5070           1 -----CGCGACCUCAGAUCAGACGUGGCGACCCGCUGAAUUUAAGCAUAUUAGUCAGCGGAGGAGA 61
28SRNA:CR45844/1-3970     1 UUAUAUACAACCUCAACUCAUAUGGGACUACCCCCUGAAUUUAAGCAUAUUAAUUAGGGGAGGAAA 66

RNA28SN5/1-5070          62 AGAAACUAACCAGGAUUCCCUCAGUAACGGCGAGUGAACAGGGAAGAGCCCAGCGCCGAAUCCCCG 127
28SRNA:CR45844/1-3970    67 AGAAACUAACAAGGAUUUUCUUAGUAGCGGCGAGCGAAAAGAAAACAGUUCAGCACUAAGUCACUU 132

RNA28SN5/1-5070         128 CCCCGCGGCGGGGCGCGGGACAUGUGGCGUACGGAAGACCCGCUCCCCGGCGCCGCUCGUGGGGGG 193
28SRNA:CR45844/1-3970   133 UGUCUAUAUGGCAAAUGUGAGAUGCAGUGUAUGGAGCGUCAAUAUUCUAGUAUGAGAAAUUAACGA 198

RNA28SN5/1-5070         194 CCCAAGUCCUUCUGAUC-GAGGCCC--AGCCCGUGGACGGUGUGAGGCCGGUAGCGGCCCCCGGCG 256
28SRNA:CR45844/1-3970   199 UUUAAGUCCUUCUUAAAUGAGGCCAUUUACCCAUAGAGGGUGCCAGGCCCGUAUAACGUUAAUGAU 264

RNA28SN5/1-5070         257 CGCCGGGCCCGGGUCUUCCCGGAGUCGGGUUGCUUGGGAAUGCAGCCCAAAGCGGGUGGUAAACUC 322
28SRNA:CR45844/1-3970   265 U-ACUAGAUGAUGUUUCCAAAGAGUCGUGUUGCUUGAUAGUGCAGCACUAAGUGGGUGGUAAACUC 329

RNA28SN5/1-5070         323 CAUCUAAGGCUAAAUACCGGCACGAGACCGAUAGUCAACAAGUACCGUAAGGGAAAGUUGAAAAGA 388
28SRNA:CR45844/1-3970   330 CAUCUAAAACUAAAUAUAACCAUGAGACCGAUAGUAAACAAGUACCGUGAGGGAAAGUUGAAAAGA 395

RNA28SN5/1-5070         389 ACUUUGAAGAGAGAGUUCAAGAGGGCGUGAAACCGUUAAGAGGUAAACGGGUGGGGUCCGCGCAGU 454
28SRNA:CR45844/1-3970   396 ACUCUGAAUAGAGAGUUAAACAGUACGUGAAACUGCUUAGAGGUUAAGCCCGAUGAACCUGAAUAU 461

RNA28SN5/1-5070         455 CCG-CCCGGAGGAUUCAACCCGGCGGCGGGUCCGGCCGUGUCGGCGGCCCGGCGGAUCUUUCCCGC 519
28SRNA:CR45844/1-3970   462 CCGUUAUGGAAAAUUCAUCAUUAAAA-----------UUG------------UAAUAUUUAA--- 500

RNA28SN5/1-5070         520 CCCCCGUUCCUCCCGACCCCUCCACCCGCCCUCCCUUCCCCCGCCGCCCCUCCUCCUCCUCCCCGG 585
28SRNA:CR45844/1-3970   501 -----------------------------------------------------AUAAUAUUAUGAGA 514

RNA28SN5/1-5070         586 AGGGGGCGGGCUCCGGCGGGUGCGGGGGUGGGCGGGCGGGGCCGGGGGUGGGGUCGGCGGGGGACC 651
28SRNA:CR45844/1-3970   515 A----------------------------------------------------------------- 515

RNA28SN5/1-5070         652 GUCCCCCGACCGGCGACCGGCCGCCGCCGGGCGCAUUUCCACCGCGGCGGUGCGCCGCGACC---- 713
28SRNA:CR45844/1-3970   516 ---------------------------UAGUGUGCAUUUUUUCCAUAUAAGGACAUUGUAAUCUAUU 555

RNA28SN5/1-5070         714 ---GGCUCCGGGACGGCUGGGAAGGCCCGGCGGGGAAGGUGGCUCGGGGGGCCCCGUCCGUCCGUC 776
28SRNA:CR45844/1-3970   556 AGCAUAUACCAAAUUUAUC------------AUAAAAUAUAACUUAUA------------------ 591

RNA28SN5/1-5070         777 CGUCCGUCCUCCUCCUCCCCCGUCUCCGCCCCCCGGCCCCGCGUCCUCCCUCGGGAGGGCGCGCGG 842
28SRNA:CR45844/1-3970   592 ----------------------GUUU------------------AUUCCAAUUAAAUUGCUUG--- 614

RNA28SN5/1-5070         843 GUCGGGGCGGCGGCGGCGGCGGCGGUGGCGGCGGCGGCGGCGGCGGGACCGAAACCCCCCCCG 908
28SRNA:CR45844/1-3970   615 ---------------------------------------------------CAUUUUAACACAGAAUA 631

RNA28SN5/1-5070         909 AGUGUUACAGCCCCCCCGGCAGCAGCACUCGCCGAAUCCCGGGGCCGAGGGAGCGAGACCCGUCGC 974
28SRNA:CR45844/1-3970   632 AAUGUUAUUA----------------AUUUGAUAAA-----GUGCUGAUAGAUUUAUAUGA----- 671

RNA28SN5/1-5070         975 CGCGCUCUCCCCCCUCCCGGCGCCCACCCCCGCGGGGAAUCCCCCGCGAGGGGGGUCUCCCCCGCG 1040
28SRNA:CR45844/1-3970   672 --------------UUACAGUG----------CG-UUAAUUUUUC--------------------- 691

RNA28SN5/1-5070        1041 GGGGCGCGCCGGCGUCUCCUCGUGGGGGGGCCGGGCCACCCCUCCCACGGCGCGACCGCUCUCCCA 1106
28SRNA:CR45844/1-3970   692 ----------GGAAU------------------------UAUAUAAUGGCAU-------------- 709

RNA28SN5/1-5070        1107 CCCCUCCUCCCCGCGCCCCCGCCCCGGCGACGGGGGGGGUGCCGCGCGCGGGUCGGGGGCGGGGC 1172
28SRNA:CR45844/1-3970       ------------------------------------------------------------------

RNA28SN5/1-5070        1173 GGACUGUCCCCAGUGCGCCCCGGGCGGGUCGCGCCGUCGGGCCCGGGGGAGGUUCUCUCGGGGCCA 1238
28SRNA:CR45844/1-3970   710 -AAUUAU---CAUUGAU----------------------------------UUUUGUGUUUAUUAUA 738

RNA28SN5/1-5070        1239 CGCGCGCGUCCCCCGAAGAGGGGGACGGCGGAGCGAGCGCACGGGGUCGGCGGCGACGUCGGCUAC 1304
28SRNA:CR45844/1-3970   739 UGCACUUGUAUGAUUAACAAUGCGA---------------------------AAGAUUCAGGAUAC 777

RNA28SN5/1-5070        1305 CCACCCGACCCGUCUUGAAACACGGACCAAGGAGUCUAACACGUGCGCGAGUCGGGGGCUCGCACG 1370
28SRNA:CR45844/1-3970   778 CUUCGGGACCCGUCUUGAAACACGGACCAAGGAGUCUAACAUAUGUGCAAGUUAUUGGG----AU- 838

RNA28SN5/1-5070        1371 AAAGCCGCCGUGGCGCAAUGAAGGUGAAGGCCGGCGCGCUCGCCGGCCGAGG-------------- 1422
28SRNA:CR45844/1-3970   839 AUAAACCUAAUAGCGUAAUUAACUUGACUAAUAAUGGGAUUAGUUUUUUAGCUAUUUAUAGCUAAU 904

RNA28SN5/1-5070        1423 ---UGGGAUCCCGAGGCCUC---UCCAGUCCG-CCGAGGGCGCACCACCGGCCCGUCUCGCCCGCC 1481
28SRNA:CR45844/1-3970   905 UAACACAAUCCCGGGGCGUUCUAUAUAGUUAUGUAUAAUGUAUAUU--UAUAUUAUUUA---UGCC 965

RNA28SN5/1-5070        1482 GC-GCCGGGGAGGUGGAGCACGAGCGCACGUGUUAGGACCCGAAAGAUGGUGAACUAUGCCUGGGC 1546
28SRNA:CR45844/1-3970   966 UCUAACUGGAA--CGUACCUUGAGCAUAUAUGCUGUGACCCGAAAGAUGGUGAACUAUACUUGAUC 1029

RNA28SN5/1-5070        1547 AGGGCGAAGCCAGAGGAAACUCUGGUGGAGGUCCGUAGCGGUCCUGACGUGCAAAUCGGUCGUCCG 1612
28SRNA:CR45844/1-3970  1030 AGGUUGAAGUCAGGGGAAACCCUGAUGGAAGACCGAAACAGUUCUGACGUGCAAAUCGAUUGUCAG 1095

RNA28SN5/1-5070        1613 ACCUGGGUAUAGGGGCGAAAGACUAAUCGAACCAUCUAGUAGCUGGUUCCCUCCGAAGUUUCCCUC 1678
28SRNA:CR45844/1-3970  1096 AAUUGAGUAUAGGGGCGAAAGACCAAUCGAACCAUCUAGUAGCUGGUUCCUUCCGAAGUUUCCCUC 1161

RNA28SN5/1-5070        1679 AGGAUAGCUGGCGCUCUCGCAGACCCGACGCACCCCCGCCACGCAGUUUUAUCCGGUAAAGCGAAU 1744
28SRNA:CR45844/1-3970  1162 AGGAUAGCUGGUGCAUUUUAAUAUUAUAU----------AAAAUAAUCUUAUCUGGUAAAGCGAAU 1217

RNA28SN5/1-5070        1745 GAUUAGAGGUCUUGGGGCCGAAACGAUCUCAACCUAUUCUCAAACUUUAAAUGGGUAAGAAGCCCG 1810
28SRNA:CR45844/1-3970  1218 GAUUAGAGGCCUUAGGGUCGAAACGAUCUUAACCUAUUCUCAAACUUUAAAUGGGUAAGAACCUUA 1283

RNA28SN5/1-5070        1811 GCUCGCUGGCG-UGGAGCCG--GGCGUGGAAUGCGAGUGCCUAGUGGGCCACUUUUGGUAAGCAGA 1873
28SRNA:CR45844/1-3970  1284 ACUUUCUUGAUAUGAAGUUCAAGGUUAUGAUAUAAUGUGCCCAGUGGGCCACUUUUGGUAAGCAGA 1349

RNA28SN5/1-5070        1874 ACUGGCGCUGCGGGAUGAACCGAACGCCGGGUUAAGGCGCCCGAUGCCGACGCUCAU-CAGACCCC 1938
28SRNA:CR45844/1-3970  1350 ACUGGCGCUGUGGGAUGAACCAAACGUAAUGUUACGGUGCCCAAAUUAACAACUCAUGCAGAUACC 1415

RNA28SN5/1-5070        1939 AGAAAAGGUGUUGGUUGAUAUAGACAGCAGGACGGUGGCCAUGGAAGUCGGAAUCCGCUAAGGAGU 2004
28SRNA:CR45844/1-3970  1416 AUGAAAGGCGUUGGUUGCUUAAAACAGCAGGACGGUGAUCAUGGAAGUCGAAAUCCGCUAAGGAGU 1481
```

```
RNA28SN5/1-5070        2005 GUGUAACAACUCACCUGCCGAAUCAACUAGCCCUGAAAAUGGAUGGCGCUGGAGCGUCGGCCCAU 2070
28SRNA:CR45844/1-3970  1482 GUGUAACAACUCACCUGCCGAAGCAACUAGCCCUUAAAAUGGAUGGCGCUUAAGUUGUAUACCUAU 1547

RNA28SN5/1-5070        2071 ACCCGGCCGUCGCCGGCAGUCGAGAGUGGACGGGAGCGGCGGGGGCGGCGCGCGCGCGCGCGUG 2136
28SRNA:CR45844/1-3970  1548 ACAUUAC------CGCU-----AAAGUAGAUGAUUUA--------------------UAUUACUUG 1582

RNA28SN5/1-5070        2137 UGGUGUGCGUCGGAGGGCGGCGGCGGCGGCGGCGGCGGGGGUGUGGGGUCCUUCCCCCGCCCCCCC 2202
28SRNA:CR45844/1-3970  1583 UGAUAUAAAUUU------------------------------------------------------ 1594

RNA28SN5/1-5070        2203 CCCCACGCCUCCUCCCCUCCUCCCGCCCACGCCCCGCUCCCCGCCCCCGGAGCCCCGCGGACGCUA 2268
28SRNA:CR45844/1-3970  1595 ----------------------------------------------------------UGA----- 1597

RNA28SN5/1-5070        2269 CGCCGCGACGAGUAGGAGGGCCGCUGCGGUGAGCCUUGAAGCCUAGGGCGCGGGCCCGGGUGGAGC 2334
28SRNA:CR45844/1-3970  1598 AACUUUAGUGAGUAGGAAGGU-ACAAUGGUAUGCGUAGAAGUGUUUGGCGUAAGCCUGCAUGGAGC 1662

RNA28SN5/1-5070        2335 CGCCGCAGGUGCAGAUCUUGGUGGUAGUAGCAAAUAUUCAAACGAGAACUUUGAAGGCCGAAGUGG 2400
28SRNA:CR45844/1-3970  1663 UGCCAUUGGUACAGAUCUUGGUGGUAGUAGCAAAUAAUCGAAUGAGACCUUGGAGGACUGAAGUGG 1728

RNA28SN5/1-5070        2401 AGAAGGGUUCCAUGUGAACAGCAGUUGAACAUGGGUCAGUCGGUCCUGAGAGAUGGGCGAGCGCCG 2466
28SRNA:CR45844/1-3970  1729 AGAAGGGUUUCGUGUGAACAGUGGUUGAUCACGAGUUAGUCGGUCCUAAGUUCAAGGCGAAAGCCG 1794

RNA28SN5/1-5070        2467 U----UCCGAAGGGACGGGCGAUGGCCU----------------------------CCGUUGCCCU 2500
28SRNA:CR45844/1-3970  1795 AAAAUUUUCAAGUAAAACAAAAAUGCCUAACUAUAUAAACAAAGCGAAUUAUAAUACACUUGAAUA 1860

RNA28SN5/1-5070        2501 CGGCCGAUCGAAAGGGAGUCGGGUUCAGAUCCCCGAAUCCGG--AGUGGC-----------GGAGA 2553
28SRNA:CR45844/1-3970  1861 AUUUUGAACGAAAGGGAAUACGGUUCCAAUUCCGUAACCUGUUGAGUAUCCGUUUGUUAUUAAAUA 1926

RNA28SN5/1-5070        2554 UGGGCGCCGCGAGGCGUCCAGUGCGGUAACGCGACCGAUCCCGGAGAAGCCGGCGGGAGCCCCGGG 2619
28SRNA:CR45844/1-3970  1927 UGGGCCUCG-----UGCUCAUCCUGGCAACAGGAACGACCAUAAAGAAGCCGUCGAGAGAUAUCGG 1987

RNA28SN5/1-5070        2620 GAGAGUUCUCUUUUCUUUGUGAAGGGCAGGGCGCCCUGGAAUGGGUUCGCCCCGAGAGAGGGGCCC 2685
28SRNA:CR45844/1-3970  1988 AAGAGUUUUCUUUUCUGUUUUAUAGCCGUACUACCAUGGAAGUCUUUCGCAGAGAGAUAUGGUAGA 2053

RNA28SN5/1-5070        2686 GUGCCUUGGAAAGCGUCGCGGUUCCGGCGGCGUCCGGUGAGCUCUCGCUGGCCCUUGAAAAUCCGG 2751
28SRNA:CR45844/1-3970  2054 UGGGCUAGAAGAGCAUGACAUAUACUGUUGUGUC-GAUAUUUUCUCCUCGGACCUUGAAAAUUUAU 2118

RNA28SN5/1-5070        2752 GGG-AGAGGGUGUAAAUCUCGCGCCGGGCCGUACCCAUAUCCGCAGCAGGUCUCCAAGGUGAACAG 2816
28SRNA:CR45844/1-3970  2119 GGUGGGGACACGCAAACUUCUCAACAGGCCGUACCAAUAUCCGCAGCUGGUCUCCAAGGUGAAGAG 2184

RNA28SN5/1-5070        2817 CCUCUGGCAUGUUGGAACAAUGUAGGUAAGGGAAGUCGGCAAGCCGGAUCCGUAACUUCGGGAUAA 2882
28SRNA:CR45844/1-3970  2185 UCUCUAGUC-GAUAGAAUAAUGUAGGUAAGGGAAGUCGGCAAAUUAGAUCCGUAACUUCGGGAUAA 2249

RNA28SN5/1-5070        2883 GGAUUGGCUCUAAGGGCUGGGUCGGUCGGGCUGGGGCGCGAAGCGGGGCUGGGCGCGCGCCGCGGC 2948
28SRNA:CR45844/1-3970  2250 GGAUUGGCUCUGAAGAUUGAGAUAGUCGGGCUUGAUUGGGAAACAAUAACA--------------- 2300

RNA28SN5/1-5070        2949 UGGACGAGGCGCCGCCGCCCCCCCCACGCCCGGGGCACCCCCCUCGCGGCCCUCCCCCGCCCCACC 3014
28SRNA:CR45844/1-3970  2301 UGGUUUAUGUGC--------------UCGUUCUGGGUAAA----UAG------------------- 2329

RNA28SN5/1-5070        3015 CCGCGCGCGCCGCUCGCUCCCUCCCCGCCCCGCGCCCUCUCUCUCUCUCUCUCCCCCGCUCCCCGU 3080
28SRNA:CR45844/1-3970  2330 -------AGUUUCUA------------------G-CAUUUAU----GUUAGUUACUUGUU------ 2359

RNA28SN5/1-5070        3081 CCUCCCCCCUCCCCGGGGGAGCGCCGCGUGGGGGCGGCGGCGGGGGAGAAGGGUCGGGGCGGCAG 3146
28SRNA:CR45844/1-3970  2360 -----------CCCCG-GAUAGUUU--A-------------------------------------- 2373

RNA28SN5/1-5070        3147 GGGCCGGCGGCGGCCCGCCGCGGGGCCCCGGCGGCGGGGGCACGGUCCCCCGCGAGGGGGGCCCGG 3212
28SRNA:CR45844/1-3970       ------------------------------------------------------------------

RNA28SN5/1-5070        3213 GCACCCGGGGGGCCGGCGGCGGCGGCGACUCUGGACGCGAGCCGGGCCCUUCCCGUGGAUCGCCCC 3278
28SRNA:CR45844/1-3970  2374 ----------------GUUACGUAGCCAAUUGUGGAA-----------CUUUCUG---------- 2402

RNA28SN5/1-5070        3279 AGCUGCGGCGGGCGUCGCGGCCGCCCCCGGGGAGCCCGGCGGGCGCCGGCGCGCCCCCCCCCCCAC 3344
28SRNA:CR45844/1-3970  2403 --CU-------------------------------------------------------------- 2404

RNA28SN5/1-5070        3345 CCCACGUCUCGUCGCGCGCGCGUCCGCUGGGGGCGGGGAGCGGUCGGGCGGCGGCGGUCGGCGGGC 3410
28SRNA:CR45844/1-3970  2405 --AAAAUUUUUAA------GAAUACUAU---------------UUGGG------------------ 2429

RNA28SN5/1-5070        3411 GGCGGGGCGGGGCGGUUCGUCCCCCCGCCCUACCCCCCCGGCCCCGUCCGCCCCCCGUUCCCCCCU 3476
28SRNA:CR45844/1-3970  2430 ------UUAAACCAAUUAGU--------------------------------------U----CUU 2447

RNA28SN5/1-5070        3477 CCUCCUCGGCGCGCGGCGGCGGCGGCGGCAGGCGGCGGAGGGGCCGCGGGCCGGUCCCCCCCGCCG 3542
28SRNA:CR45844/1-3970  2448 A----------------------------------------------------------------- 2448

RNA28SN5/1-5070        3543 GGUCCGCCCCCGGGGCCGCGGUUCCGCGCGGCGCCUCGCCUCGGCCGGCGCCUAGCAGCCGACUUA 3608
28SRNA:CR45844/1-3970  2449 ---------------------UUA----------AUUAUAACGAUUAUCAAUUAACAAUCAAUUCA 2483

RNA28SN5/1-5070        3609 GAACUGGUGCGGACCAGGGGAAUCCGACUGUUUAAUUAAAACAAAGCAUCGCGAAGGCCCGCGGCG 3674
28SRNA:CR45844/1-3970  2484 GAACUGGCACGGACUUGGGGAAUCCGACUGUCUAAUUAAAACAAAGCAUUGUGAUGGCCCU-AGCG 2548

RNA28SN5/1-5070        3675 GGUGUUGACGCGAUGUGAUUUCUGCCCAGUGCUCUGAAUGUCAAAGUGAAGAAAUUCAAUGAAGCG 3740
28SRNA:CR45844/1-3970  2549 GGUGUUGACACAAUGUGAUUUCUGCCCAGUGCUCUGAAUGUCAAAGUGAAGAAAUUCAAGUAAGCG 2614

RNA28SN5/1-5070        3741 CGGGUAAACGGCGGGAGUAACUAUGACUCUCUUAAGGUAGCCAAAUGCCUCGUCAUCUAAUUAGUG 3806
28SRNA:CR45844/1-3970  2615 CGGGUCAACGGCGGGAGUAACUAUGACUCUCUUAAGGUAGCCAAAUGCCUCGUCAUCUAAUUAGUG 2680

RNA28SN5/1-5070        3807 ACGCGCAUGAAUGGAUGAACGAGAUUCCCAC-UGUCCCUACCUACUAUCCAGCGAAACCACAGCCA 3871
28SRNA:CR45844/1-3970  2681 ACGCGCAUGAAUGGAUUAACGAGAUUCCUACUUGUCCCUAUCUACUAUCUAGCGAAACCACAGCCA 2746

RNA28SN5/1-5070        3872 AGGGAACGGGCUUGGCGGAAUCAGCGGGGAAAGAAGACCCUGUUGAGCUUGACUCUAGUCUGGCAC 3937
28SRNA:CR45844/1-3970  2747 AGGGAACGGGCUUGGAAUAAUUAGCGGGGAAAGAAGACCCUUUUGAGCUUGACUCUAAUCUGGCAG 2812

RNA28SN5/1-5070        3938 GGUGAAGAGACAUGAGAGGUGUAGAAUAAGUGGGAGGCCCCCGGCGCCCCCCCGGUGUCCCCGCGA 4003
28SRNA:CR45844/1-3970  2813 UGUAAGGAGACAUAAGAGGUGUAGAAUAAGUGGGAGAUAUUAGACCU-----CGGU---------- 2863
```

```
RNA28SN5/1-5070           4004 GGGGCCCGGGGCGGGGUCCGCCGGCCCUGCGGGCCGCCGGUGAAAUACCACUACUCUGAUCGUUUU 4069
28SRNA:CR45844/1-3970     2864 ---------------------------UUGGUAUCGUCAAUGAAAUACCACUACUCUUAUUGUUUC 2902

RNA28SN5/1-5070           4070 UUCACUGACCCGGUGAGGCGGGGGGGC----------GAGCCCGAG-------------------- 4106
28SRNA:CR45844/1-3970     2903 CUUACUUACUUGAUUAAAUGGAACGUGUAUCAUUUCCUAGCCAUUAUACGGAUAUAUUUAUUAUAU 2968

RNA28SN5/1-5070           4107 -----G-----GGCU-------CUCGCUUCUGGCGCCAAGCGCCCGG------------------- 4136
28SRNA:CR45844/1-3970     2969 CUUAUGGUAUUGGGUUUUGAUGCAAGCUUCUUGAUCAAAGUAUCACGAGUUUGUUAUAUAAUCGCA 3034

RNA28SN5/1-5070           4137 ------------------------------------------------------C----CGCGCGC 4144
28SRNA:CR45844/1-3970     3035 AACAAAUUCUUUAAUAAAACGAUGCAUUUAUGUAUUUUUGAUUUGAAAAUUUGGUAUAACUCCAAU 3100

RNA28SN5/1-5070           4145 CGGCCGGGCGCGACCCGCUCCGGGGACAGUGCCAGGUGGGGAGUUUGACUGGGGCGGUACACCUGU 4210
28SRNA:CR45844/1-3970     3101 UACUCAGGUAUGAUCCAAUUCAAGGACAUUGCCAGGUAGGGAGUUUGACUGGGGCGGUACAUCUCU 3166

RNA28SN5/1-5070           4211 CAAACGGUAACGCAGGUGUCCUAAGGCGAGCUCAGGGAGGACAGAAACCUCCCGUGGAGCAGAAGG 4276
28SRNA:CR45844/1-3970     3167 CAAAUAAUAACGGAGGUGUCCCAAGGCCAGCUCAGUGCGGACAGAAACCACACAUAGAGCAAAAGG 3232

RNA28SN5/1-5070           4277 GCAAAAGCUCGCUUGAUCUUGAUUUUCAGUACGAAUACAGACCGUGAAAGCGGGGCCUCACGAUCC 4342
28SRNA:CR45844/1-3970     3233 GCAAAUGCUGACUUGAUCUCGGUGUUCAGUACACACAGGGACAGCAAAAGCUCGGCCUAUCGAUCC 3298

RNA28SN5/1-5070           4343 UUCUGACCUUUUGGGUUUUAAGCAGGAGGUGUCAGAAAAGUUACCACAGGGAUAACUGGCUUGUGG 4408
28SRNA:CR45844/1-3970     3299 UUUUGGUUUAAAGAGUUUUUAACAAGAGGUGUCAGAAAAGUUACCAUAGGGAUAACUGGCUUGUGG 3364

RNA28SN5/1-5070           4409 CGGCCAAGCGUUCAUAGCGACGUCGCUUUUUGAUCCUUCGAUGUCGGCUCUUCCUAUCAUUGUGAA 4474
28SRNA:CR45844/1-3970     3365 CGGCCAAGCGUUCAUAGCGACGUCGCUUUUUGAUCCUUCGAUGUCGGCUCUUCCUAUCAUUGUGAA 3430

RNA28SN5/1-5070           4475 GCAGAAUUCACCAAGCGUUGGAUUGUUCACCCACUAAUAGGGAACGUGAGCUGGGUUUAGACCGUC 4540
28SRNA:CR45844/1-3970     3431 GCAAAAUUCACCAAGCGUUGGAUUGUUCACCCAUG-CAAGGGAACGUGAGCUGGGUUUAGACCGUC 3495

RNA28SN5/1-5070           4541 GUGAGACAGGUUAGUUUUACCCUACUGAUGAU---GUGUUGUUGCCAUGGUAAUCCUGCUCAGUAC 4603
28SRNA:CR45844/1-3970     3496 GUGAGACAGGUUAGUUUUACCCUACUAAUGACAAAACGUUGUUGCGACAGCAUUCCUGCGUAGUAC 3561

RNA28SN5/1-5070           4604 GAGAGGAACCGCAGGUUCAGACAUUUGGUGUAUGUGCUUGGCUGAGGAGCCAAUGGGGCGAAGCUA 4669
28SRNA:CR45844/1-3970     3562 GAGAGGAACCGCAGGUACGGACCAAUGGCACA-AUACUUGUUCGAGCGAACAGUGGUAUGACGCUA 3626

RNA28SN5/1-5070           4670 CCAUCUGUGGGAUUAUGACUGAACGCCUCUAAGUCAGAAUCCCGCCCAGG-CGGAACGAUACGGCA 4734
28SRNA:CR45844/1-3970     3627 C-GUCCGUUGGAUUAUGCCUGAACGCCUCUAAGGUCGUAUCCGUGCUGGACUGCAAUGAUAAAUAA 3691

RNA28SN5/1-5070           4735 GCGCCGCGGAGCCUCGGUUGGCCUCGGAUAGCCGGUCCCCCGCCUGUCCCCGCCGGCGGGCCGCCC 4800
28SRNA:CR45844/1-3970     3692 GGGGCAA----------UUUGCAUUGUAUGGCUUCUAAACCAUUUAA---AGUUUA------UAAU 3738

RNA28SN5/1-5070           4801 CCCCCCUCCACGCGCCCCGCGCGCGCGGGAGGGCGCGUGCCCCGCCGCGCGCCGGGACCGGGGUCC 4866
28SRNA:CR45844/1-3970     3739 UUACUUUAUAAACGAC-------------AAU----------------------------GGAUGU 3763

RNA28SN5/1-5070           4867 GGUGCGGAGUGCCCUUCG--UCCUGGGAAACGGGGCGCGGCC-GGAGAGGCGGCCGCCCCCUCGCC 4929
28SRNA:CR45844/1-3970     3764 GAUGCC-AAUGUAAUUUGUAACAUAGUAAAUUGGGAGGAUCUUCGAU---CACCUGAUGCCGCGCU 3825

RNA28SN5/1-5070           4930 CGUCACGCACCGCACG-UUCGUGGG-----GAACCUGGCGCUAAACCAUUCGUAGACGACCUGCUU 4989
28SRNA:CR45844/1-3970     3826 AGUUACAUAUAAAAGCAUUAUUUAAUACAAUGACAAAGCCUAGAAUCAAUUGUAAACGACUUUUGU 3891

RNA28SN5/1-5070           4990 CUGGGUCGGGGUUUCGUACGUAGCAGAGCAGCUCCCUCGCUGCGAUCUAUUGAAAGUCAGCCCUCG 5055
28SRNA:CR45844/1-3970     3892 AACAGGCAAGGUGUUGUAAGUGGUUGAGCAGCUGCCAUACUGCGAUCCACUGAAGCUUAUCCUUUG 3957

RNA28SN5/1-5070           5056 ACACAAGGGUUUGUC                                                    5070
28SRNA:CR45844/1-3970     3958 CUUGAU-GAUUCGA-                                                    3970
```
